# Phytochemistry, Pharmacological Potential, and Future Prospects of Abietane‐Type Diterpenoids From *Salvia rosmarinus*

**DOI:** 10.1002/cbdv.71841

**Published:** 2026-10-12

**Authors:** Rida Siddiqui, Vipin Singh, Kapil Dev

**Affiliations:** ^1^ Phytochemistry Division CSIR‐ Central Institute of Medicinal and Aromatic Plants Lucknow India; ^2^ Academy of Scientific and Innovative Research (AcSIR) Ghaziabad India

**Keywords:** abietane‐type diterpenoids, geranylgeranyl diphosphate, rosemary, *Salvia*, *Salvia rosmarinus*

## Abstract

*Salvia rosmarinus*, formerly known as *Rosmarinus officinalis*, is a medicinal plant known as one of the sources of abietane‐type diterpenoids. The largest family of chemical constituents from the *Salvia* genus is abietanes and rearranged abietanes. Since 1976, 365 of the 545 known diterpenoids have been recognized as abietane diterpenoids. Carnosic acid, carnosol, rosmanol, epirosmanol, and their derivatives are among the bioactive abietanes that have gained much attention because of the wide spectrum of pharmacological activities. This review presents information on the distribution, isolation, biosynthesis, structural variation, degradation, semi‐synthetic strategies, pharmacokinetics, and bioactivities of abietane‐type diterpenoids from *S. rosmarinus*. Recent developments in eco‐friendly extraction techniques, namely, supercritical fluid extraction, microwave‐assisted extraction, ultrasound‐assisted extraction, and pressurized liquid extraction, are also described in this review. The biosynthetic pathway from geranylgeranyl diphosphate to main diterpenoids, such as carnosic acid and carnosol, is also presented. Moreover, newly isolated compounds, structure‐activity relationships, and antioxidant, antimicrobial, anti‐inflammatory, neuroprotective, and anticancer activities are included. Moreover, the pharmacokinetics of abietane diterpenoids isolated from *S. rosmarinus* and their medicinal applications are also covered in this review.

## Graphical abstract

The review delves into the findings on abietane diterpenes from *Salvia rosmarinus*. It comprises the phytochemistry, biological activities, biosynthesis, degradation studies, semisynthesis, and pharmacokinetic studies of these compounds. The potential of these compounds for future development has also been highlighted.

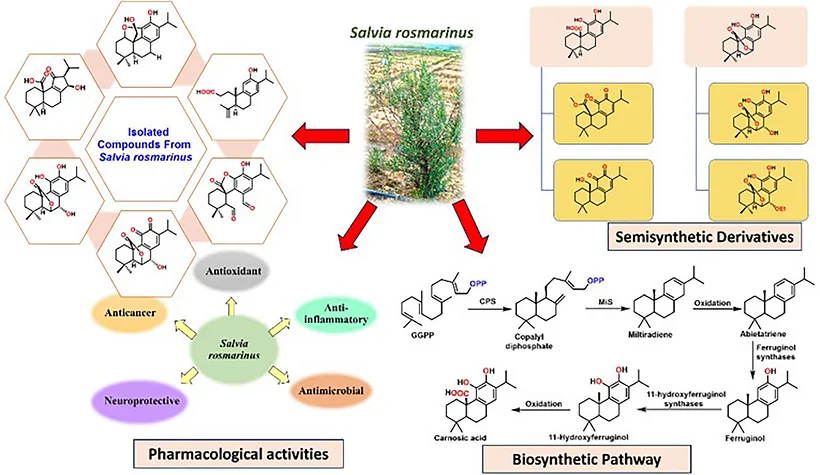

**Abbreviations**
AKT: protein Kinase B
ARE: antioxidant response element
AST: aminotransferase
AUC: area under the curve
Bax: bcl‐2‐associated X protein
Bcl‐2: *β*‐cell lymphoma‐2
BHA: butylated hydroxyanisole
BHT: butylated hydroxytoluene
CA: carnosic acid
CAT: catalase
CDKs: cyclin‐dependent kinases
C_max_
: maximum plasma concentration
COX: cyclooxygenase
CPS: copalyl diphosphate synthase
CS: carnosol
DAD: UV‐diode array detector
DMAPP: dimethylallyl diphosphate
EMT: epithelial mesenchymal transition
ER: endoplasmic reticulum
ERK: extracellular signal regulated kinase
FPP: farnesyl diphosphate
FPS: farnesyl diphosphate synthase
GGPP: geranylgeranyl diphosphate
GGPS: geranylgeranyl diphosphate synthase
GPP: geranyl diphosphate
GPS: geranyl diphosphate synthase
HIV: human immunodeficiency virus
HO: 1‐heme oxygenase‐1
HPLC: high‐performance liquid chromatography
IL: interleukin
iNOS: inducible nitric oxide synthase
IPP: isopentenyl pyrophosphate
JAK: Janus kinase
JNK‐c: Jun N‐terminal Kinase
Keap1: Kelch‐like ECH‐associated protein 1
LD_50_
: median lethal dose
MAE: microwave‐assisted extraction
MAPKs: mitogen‐activated protein kinases
Mdm2: mouse double minute 2
MEP: methylerythritol Phosphate
MIC: minimum inhibitory concentration
MiS: miltiradiene synthase
MMP: matrix metalloproteinases
MS: mass spectrometry
mTOR: mechanistic target of rapamycin (mTOR)
NF‐*κ*B: nuclear factor kappa‐light‐chain‐enhancer
NMR: nuclear magnetic resonance
NO: nitric oxide
Nrf2: nuclear factor‐erythroid 2‐related factor 2
PARP: poly(ADP‐ribose) polymerase
PGE2: prostaglandin E2
P‐gp: P‐glycoprotein
PI3K: phosphoinositide 3‐kinase
PLE: pressurized liquid extraction
PMNL: polymorphonuclear leukocytes
ROS: reactive oxygen species
SFE: supercritical fluid extraction
SOD: superoxide dismutase
STAT: signal transducer and activator of transcription
TIMPs: tissue inhibitors of metalloproteinases
T_max_
: time of maximum concentration
TNF: tumour necrosis factor
TOF: time of flight
TTCCs: T‐type calcium channels
UAE: ultrasound‐assisted extraction
uPA: urokinase plasminogen activator

## 1 Introduction

*Salvia* is a member of the Lamiaceae family, which is the largest in the world, with over 220 genera and 4,000 species found almost everywhere. This genus contains 900 species worldwide [1]. Its major habitats are in East Asia, Southwest Asia, the Mediterranean, and Mesoamerica/South America [2]. Abietane‐type diterpenoids are a class of compounds found in numerous plant species, particularly in this genus. Abietanes are naturally occurring diterpenoids derived from terrestrial plants with diverse origins. The range of fascinating biological activity they exhibit piques the curiosity of the pharmaceutical and medicinal industries [3]. The name of the abietane diterpenoid comes from abietic acid, which is one of the resin acids found in coniferous oleoresin. Abietic acid was discovered and thoroughly studied in the early twentieth century and serves as the basic structure of the abietane class. Later, the name “abietane” was standardized based on abietic acid [4]. The review covers the phytochemistry, pharmacological potential, and prospects for the future with regard to abietane‐type diterpenoids found in *Salvia rosmarinus*. Abietane‐type diterpenoids (Figure 1) are among the numerous bioactive compounds occurring in the genus *Salvia*, which is also known as Sage.

**FIGURE 1:**
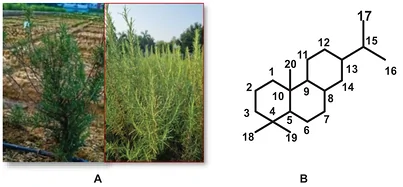
(A) *Salvia rosmarinus* plant. (B) Basic skeleton of abietane‐type diterpenes.

The diverse biological activities of abietane‐type diterpenoids in *S. rosmarinus* are due to the tricyclic diterpenoid skeleton. The most common abietane‐type diterpenoids found in *S. rosmarinus* are carnosic acid and carnosol. Rosmanol, occurring in *S. rosmarinus*, is also reported to exhibit antitumor as well as antiviral activity. Recently, six new abietane diterpenoids, rosmarinusin Q‐V (Figure 2), have also been reported from *S. rosmarinus* using phytochemical investigation. Out of these, rosmarinusin Q was a novel abietane diterpenoid containing a 6/6/5 skeleton, whose ring C was an *α, β*‐unsaturated five‐element ketone [5]. Spectroscopic techniques such as NMR and mass spectrometry are generally employed to determine the structure of these compounds. Abietanes and rearranged abietanes form the major group of *Salvia* plant constituents. It is the most abundant known group of *Salvia* diterpenoids, with 19 subgroups, and is seemingly the most distributed [6].

**FIGURE 2:**
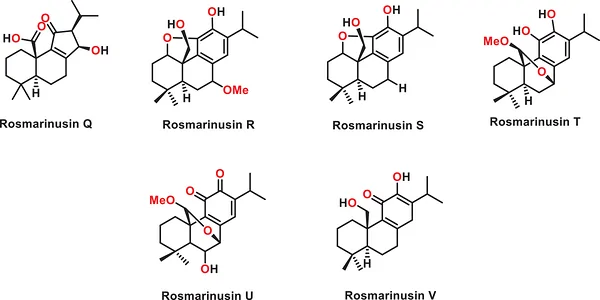
Chemical structures of compounds Rosmarinusin Q‐V.

Rosemary contains trace levels of rosmanol, but its two main and pharmacologically significant diterpenes are carnosic acid (CA) and carnosol (CS). In particular, the dried rosemary leaves contain 4 g of carnosic acid and 0.4 g of carnosol per 100 g, respectively [7, 8]. However, it also depends upon the chemotype of the species. These substances are *o*‐diphenolic diterpenes having an abietane scaffold made up of a fused six‐membered tricyclic ring structure with an aromatic ring. Their glucuronide derivatives appeared in plasma as early as 25 min after oral administration in rats, suggesting their high bioavailability [9]. More specifically, the bioavailability of CA was approximately 40% after oral administration [10]. In vivo, three major processes are responsible for the breakdown of carnosic acid: oxidation, glucuronidation, and methylation [11]. Finally, carnosic acid was detected in rat brain tissue, which suggests that it crosses the blood–brain barrier. This provides a basis for several studies that have identified neuroprotective and cognitive benefits.

## 2 Methodology

Information about the chemical composition, pharmacology, and biological activities of *S. rosmarinus* was collected using the databases Scopus, Web of Science, Google Scholar, ScienceDirect, and PubMed. The following search terms were used: “abietane diterpenoids in *Rosmarinus officinalis”*, or “abietane diterpenoids in *Salvia”*, or “abietane diterpenoids”, or “abietane diterpenoids in *S. rosmarinus*”. A total of 1838 search results were obtained from these databases, and after screening and assessment of the results, 85 references were found suitable to include in the review (Figure 3).

**FIGURE 3:**
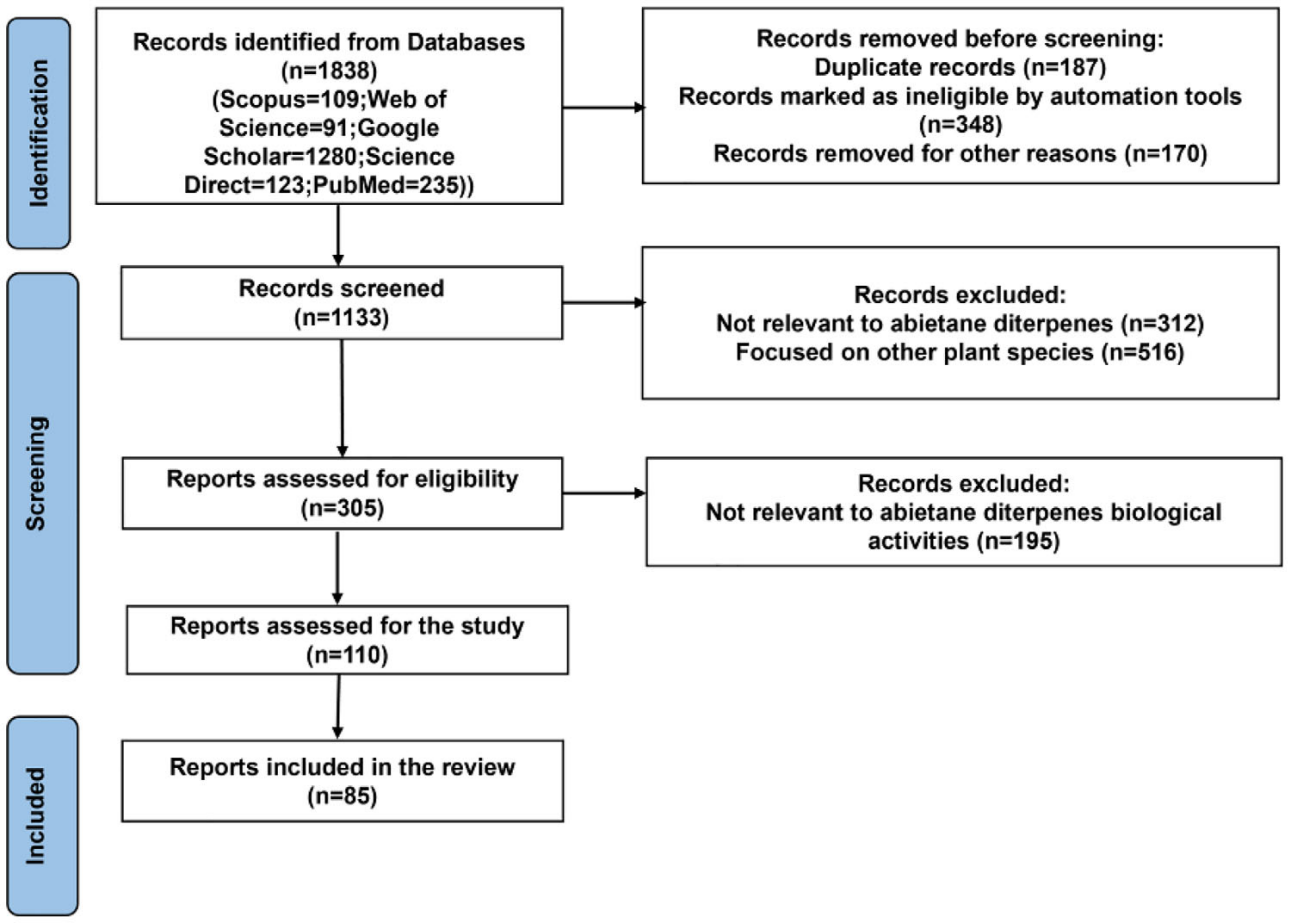
PRISMA flow diagram for the inclusion of the databases and registries in the review.

## 3 Inclusion and Exclusion Criteria

The search for this review was solely performed in the English language since 1965, reporting pharmacological and chemical data, and some experimental studies on animals, using the isolated compounds from *S. rosmarinus*. Finally, in order to ensure reliability, only publications in peer‐reviewed journals were chosen.

## 4 Historical Significance

Greeks and Romans used *S. rosmarinus* for medical purposes since 500 B.C. Even in 3000 B.C., Egyptian tombs included dried rosemary sprigs. Because of its pleasant scent, the ancient Egyptians valued rosemary. They used it in cosmetics, mummy preservation, and other rituals. In medieval Europe, people commonly used rosemary at weddings to symbolize loyalty and devotion. It also served to treat various ailments, including headaches, digestive issues, and breathing problems [12].

## 5 Morphological Distribution of Abietane‐Diterpenoids

In *Salvia* species, abietane‐type diterpenes are primarily found in the aerial portions (leaves, stems) rather than in rhizomes and roots. In numerous rosemary tissues, researchers examined the distribution of carnosic acid, 12‐*O*‐methyl carnosic acid, carnosol, rosmanol, and 11,12‐di‐*O*‐methyl isorosmanol. The relative levels of diterpenes were similar across the tissues analyzed. Carnosic acid was six times more concentrated than 12‐*O*‐methyl carnosic acid and carnosol, which were found in similar amounts. Rosmanol appeared in trace quantities, while isorosmanol showed a somewhat lower concentration than carnosol. The concentration of 11,12‐di‐*O*‐methyl isorosmanol was ten times less than that of isorosmanol. Diterpenes were not found in every tissue analyzed. Sepals had 3.2 times the diterpene level of petals, while leaves contained around 30% less. Trace amounts of diterpenes appeared in stems, and small amounts were present in seeds. Diterpenes were more abundant in the leaves compared to the roots. The oxidation product of carnosic acid, isorosmanol, undergoes O‐methylation within the chloroplasts. The resultant diterpene, 11,12‐di‐*O*‐methyl isorosmanol, is subsequently transferred by the chloroplasts to the rosemary's plasma membrane [13].

## 6 Extraction Methods of Abietane‐Diterpenoids

The extraction parameters, such as temperature, particle size, type of solvent, polarity, concentration, and acidity, all determine the efficiency of the extraction of various compounds of rosemary [14, 15]. Recently, more effective and sustainable methods have been employed for the extraction of bioactive compounds from rosemary [16]. High temperatures during processing result in the loss of bioactivity of the extracts due to the fact that rosemary contains mostly thermolabile compounds. Therefore, low temperatures during extraction are favorable [17]. The quest for natural alternatives with a wide range of applications fuels the extraction of rosemary's bioactive compounds, which are a fundamental component of scientific research [14, 15, 18].

The extraction of bioactive components from rosemary has recently moved toward more sustainable and efficient methods [16]. Maceration, decoction, conventional reflux, and Soxhlet extraction are some examples of traditional extraction methods that have been widely used [19]. Maceration and Soxhlet extraction are two conventional methods of extraction that were commonly used in the past for preparing extracts of nonvolatile compounds. However, owing to the many disadvantages associated with these methods, such as the long time required for extraction, thermal degradation of thermolabile compounds, high energy and solvent requirements, contamination of the extract, and loss of solute, these methods are rarely employed in an industrial environment.

Currently, a number of new and promising eco‐friendly bioactive extract production methods from rosemary are under investigation. Supercritical fluid extraction (SFE), pressurized liquid extraction (PLE), ultrasound‐assisted extraction (UAE), and microwave‐assisted extraction (MAE) are the most investigated techniques for the production of rosemary extracts. When choosing a method, cost, time, and the stability of the targeted compounds must be considered. This pairing of efficacy and history places a high premium on modifying traditional extraction methods to modern applications in the search for bioactive constituents from rosemary [20, 21]. These strategies seek to develop new applications across a variety of food storage and pharmaceutical industries, based on the full utilization of the bioactive compounds in rosemary. Identifying optimum extraction methods is in line with the general green biorefinery idea, which focuses on sustainability and resource effectiveness [22].

### 6.1 Ultrasound‐ And Microwave‐Assisted Extraction

Currently, two of the most frequently employed “green” techniques for accelerating the extraction process are MAE and UAE. Various methods have been proposed until now to efficiently extract the phenolic fraction from rosemary leaves. Bellumori M and co‐workers investigated the efficiency and selectivity of these methods in extracting specific components from dried rosemary leaves using food‐grade solvents. UAE was performed using a probe system equipped with a titanium horn operating at 19.5 kHz (140 W). MAE was performed at 100°C using a closed multimode reactor under an N_2_ atmosphere (20 bar). After dissolving each extract in a fixed volume of solvent, solutions were analyzed directly using complementary time‐of‐flight (TOF) and high‐performance liquid chromatography‐UV‐diode array detector‐mass spectrometry (HPLC–DAD–MS) methods. MAE and UAE in ethanol and acetone resulted in significant improvements in the yields of phenol, by more than three times compared to more conventional solid‐liquid extraction methods [23].

### 6.2 Supercritical Fluid Extraction

There are a number of ways that CO_2_ extraction can be used to recover oil esters from plant material. One is to combine plant oil, water, and crushed raw material in a specific ratio to create a mixed feed liquid. The plant extract oil is then created by subjecting the mixed feed liquid to supercritical CO_2_ extraction to yield extract liquor, which is then separated further from the water by oil in a high‐speed centrifuge. To yield an expanded liquid, another technique is milling of the material, ethanol addition as an assisted solvent, and subsequent expansion of the treated sample under CO_2_ pressure. This technique improves the oil extraction yield and reduces the extraction pressure by soaking the substance in liquid CO_2_ [24]. Benefits of this approach include lower energy requirements, selectivity, and faster extraction procedures [25, 26, 27]. The potential of SFE to produce bioactive compounds has also been studied extensively [28, 29].

A two‐step sequential SFE procedure was developed by del Pilar Sanchez Camargo et al. to produce rosemary extracts that are substantially enriched in carnosic acid and carnosol. The less active interfering fractions, such as waxes and oleoresins, were eliminated during the first extraction step using supercritical CO_2_ at 300 bar and 40°C after 60 min. The recovery of CA (403.04 mg/g) and CS (44.56 mg/g) was improved after 120 min during the second extraction step with CO_2_ and 7% ethanol as a modifier at 150 bar and 40°C. Compared to single‐step SFE, the proposed two‐stage SFE also decreased the overall extraction time from 300 to 180 min. It was also noted that the incorporation of a 7% ethanol modifier increased the yield of rosemary SFE extract from 3.1 to 6.5% at 100°C and 100–150 bar. The authors concluded that SFE of CO_2_ with a 7% ethanol modifier can be an effective method for extracting moderately polar compounds such as CA and CS in rosemary [28].

### 6.3 Pressurized Liquid Extraction

The most applied method to extract bioactive phenolic compounds from *S. rosmarinus* is PLE, followed by SFE. In this respect, Herrero et al. reported that PLE at high temperatures of 200°C with water and ethanol as the preferred solvents resulted in rosemary extracts with high yields of about 40% and high antioxidative activity with an EC_50_ of 8.8 µg/mL after 20 min [30].

Under these conditions, carnosic acid (52.87 mg/g) was extracted effectively. Moreover, PLE with ethanol turned out to be more successful for less polar molecules, such as CA and CS. Finally, Vázquez et al. determined that the yields of CA and CS, amounting to 161.49 and 13.64 mg/g, respectively, were twice as high in PLE extracts obtained with hexane at 100°C and 100 bar after 20 min compared to SFE extracts obtained at the same temperature and using pure supercritical CO_2_ after 5 h at 200°C and 300 bar [31].

## 7 Biosynthetic Pathway

The terpene precursors dimethylallyl diphosphate (DMAPP, C_5_) and isopentenyl diphosphate (IPP, C_5_) can be synthesized separately in the cytosol and plastid through separate pathways. The prenyltransferases mediate the formation of the terpene precursors with IPP and DMAPP. The formation of geranyl diphosphate (GPP) from geranyl diphosphate synthase (GPS) mediates the synthesis of monoterpenes (C_10_), farnesyl diphosphate (FPP) from farnesyl diphosphate synthase (FPS) mediates the synthesis of sesquiterpenes (C_15_) and triterpenes (C_30_), while geranylgeranyl diphosphate (GGPP) from geranylgeranyl diphosphate synthase (GGPS) mediates the synthesis of diterpenes (C_20_) and tetraterpenes (C_40_) [32, 33].

GGPP is the main C_20_ precursor for the biosynthesis of abietane diterpenoids in *S. rosmarinus* and acts as an important intermediate molecule responsible for the formation of unique diterpene skeletons. The general isoprenoid precursors, IPP and DMAPP, are involved in consecutive condensation reactions catalyzed by prenyltransferases to generate the C_20_ prenyl diphosphate precursor GGPP through the methylerythritol phosphate (MEP) pathway within the plastids [34]. The terpene synthases copalyl diphosphate synthase (CPS) and miltiradiene synthase (MiS), which resemble kaurene synthase, catalyze the initial committed steps of abietane diterpenoid biosynthesis. The enzyme CPS converts GGPP into copalyl diphosphate, and the latter is converted to miltiradiene by MiS. Miltiradiene spontaneously undergoes an aromatization or oxidation process, resulting in abietatriene, which acts as the immediate precursor for phenolic abietane diterpenoids in rosemary and its related species from the *Lamiaceae* family. The aromatic diterpene called abietatriene is then modified by cytochrome P450 monooxygenase enzymes, mostly from the CYP76 family. These enzymes, called CYP76AH, carry out a series of oxidations of abietatriene, yielding ferruginol and 11‐hydroxyferruginol. Enzymes CYP76AK contribute to further oxidation at the C_20_ site, catalyzing 11‐hydroxyferruginol transformation via intermediate steps that lead to the formation of carnosic acid, a major phenolic abietane diterpenoid in rosemary leaves [35, 36, 37]. Due to oxidation, hydroxylation, methylation, and rearrangement reactions, carnosic acid acts as an important biosynthetic center from which numerous structurally related abietane diterpenoids originate. Carnosol is one such abietane, synthesized by the spontaneous oxidation of carnosic acid, and it significantly increases the antioxidant properties of rosemary extracts. Thus, the GGPP route that links isoprenoid metabolism with biosynthesis of pharmaceutically valuable abietanes such as ferruginol, carnosic acid, carnosol, rosmanol, and other diterpenoid derivatives of diterpenes can be considered the fundamental biosynthetic pathway for the generation of rosemary abietane diterpenoids (Figure 4).

**FIGURE 4:**
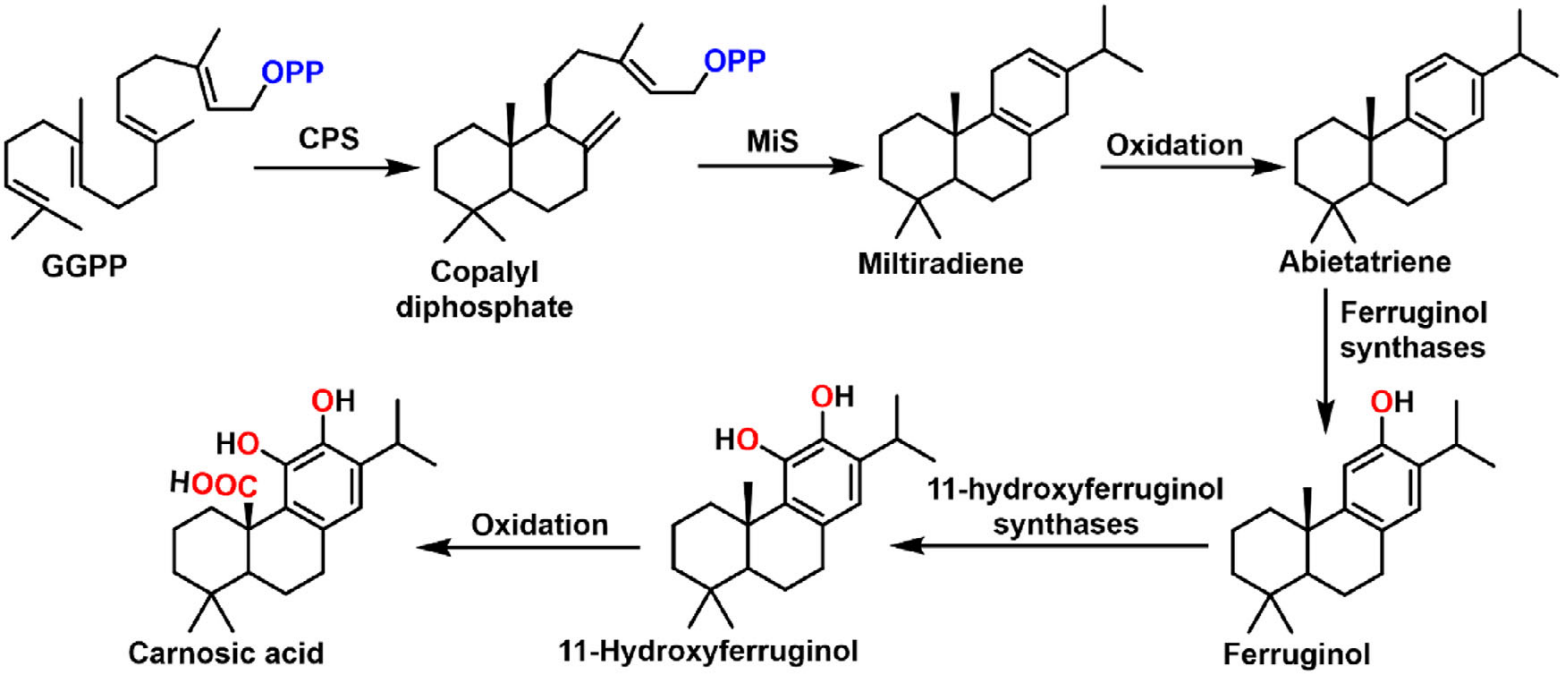
Biosynthesis of abietane‐type diterpenoids from geranylgeranyl diphosphate (GGPP).

## 8 Diterpenoids’ Structural Occurrence and Degradation Properties

In 1969, after synthesizing fichtelite, a fossil resin hydrocarbon from abietic acid (7,13‐abietadien‐18‐oic acid), Burgstahler created a semi‐systematic system for naming and numbering the diterpenoid family [38]. Substances with a 20‐carbon core structure are called aromatic abietanes [3]. A‐ring carbons typically do not provide functional groups to aromatic abietanes. Most of the B‐ and C‐ring carbons have various levels of oxidation. Usually, double bonds occur on ring C, with amounts ranging from three (abietatrienes) to four (abietatetraenes). The aromatic abietane group contains many different chemical structures derived from dehydroabietic acid. We can categorize them by the type of carbocyclic framework and the number of double bonds. These include tricyclic abietatrienes, abietatriene lactones (compounds with three double bonds and a lactone ring), and abietatetraenes (compounds with four double bonds) (Figure 5).

**FIGURE 5:**
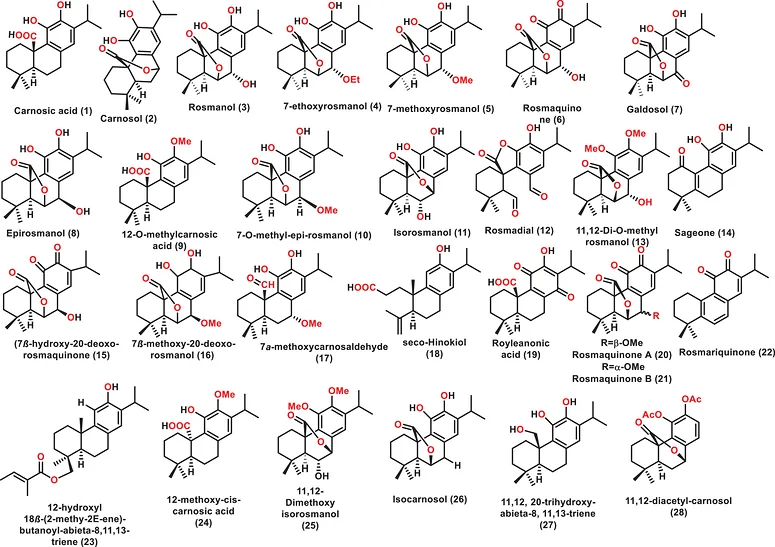
Chemical structures of abietane–type diterpenoids found in *S. rosmarinus*.

CA, a derivative of pisiferic acid, contains a C_20_ carboxylic acid. It is found in the well‐known *Lamiaceae* herb, rosemary (*S. rosmarinus*). The *o*‐diphenol structure of carnosic acid is easily oxidized and has strong antioxidant properties [36]. Researchers have investigated several biological activities of CA. For example, CA stimulates T98G human glioblastoma cells to produce nerve growth factor, suggesting potential as a treatment for Alzheimer's disease [39]. CA also prevents excitotoxicity and oxidative stress in neurons. It protects the brain from middle cerebral artery ischemia/reperfusion and increases reduced glutathione levels in vivo [40]. Studies show that CA has anticancer effects [17]. For example, in combination with turmeric or curcumin, CA strongly inhibits the growth of ER‐negative human breast cancer cells and induces G_1_ cell cycle arrest [41].

The main constituents of rosemary from the *Lamiaceae* family are lactones. These compounds are part of a group called abietatriene lactones. The other key component of these plants is that CA is oxidized to produce them. Carnosol has hydroxy groups on carbons C_11_ and C_12_, and a keto group on carbon C_20_, along with an aromatic C ring. This abietane shows antibacterial effects against oral bacteria that lead to tooth decay, as well as anti‐HIV activity (IC_50_ 8.0 µM) [42].

CS and CA suppress the formation of pro‐inflammatory leukotrienes in whole human polymorphonuclear leukocytes (PMNL) (IC_50_ 7.0 µM [CS], 15–20 µM [CA]). Anti‐inflammatory activity of CS and CA has also been investigated in vivo. Since CS has the potential to inhibit DNA damage and tumor growth in the rat mammary gland, it can be employed as a chemopreventive drug for breast cancer [43]. More oxidized diterpenes, such as 11,12‐di‐O‐methylrosmanol, isorosmanol, and rosmadial, are synthesized from rosemary leaves by the antioxidant activity of carnosic acid [8, 44]. The free radical scavenging ability of the molecule is reduced by the O‐methylation of one of the two hydroxyl groups at C_11_ and C_12_ [45].

### 8.1 Degradation Study of Abietane Diterpenoids

Carnosic acid was detected as the major phenolic diterpene in rosemary leaves, while rosmanol, epirosmanol, and 7‐methyl‐epirosmanol were only present in small amounts. This indicates that lactone‐structured phenolic diterpenes may form during extraction when the extract is exposed to air [46]. According to Wenkert et al., benzoquinone changes to semiquinone, which then transforms into *δ*‐ and *γ*‐lactones [47]. The production of carnosol led to a drop in the concentration of carnosic acid, which then decomposed to produce *γ*‐lactones, rosmanol, epirosmanol, and 7‐methyl‐epirosmanol [46]. The *γ*‐lactones, rosmanol, epirosmanol, and 7‐methyl‐epirosmanol come from carnosol instead of the quinone and semiquinone intermediates of carnosic acid (Figure 6) [48].

**FIGURE 6:**
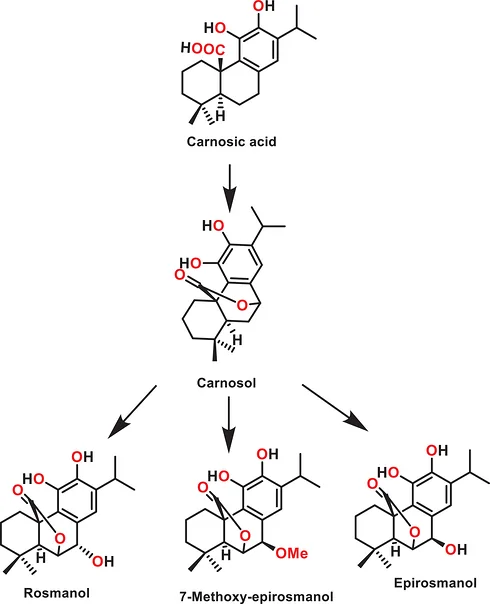
Degradation of carnosic acid to different modified diterpenoids.

## 9 Structure‐Activity Relationship

The abietane diterpenoids isolated from *S. rosmarinus* are carnosic acid, carnosol, rosmanol, epirosmanol, isorosmanol, and galdosol. Some structural features of the abietane framework play a key role in influencing the biological activities of these compounds, including antioxidant, antibacterial, anti‐inflammatory, and anticancer activities.

Similar to carnosic acid and its derivatives, having a hydroxyl group at positions C_11_ and C_12,_ and a carboxyl group at position C_20_ gives rise to antitumor compounds. Compounds containing catechol, such as carnosol and its derivatives, with a diosphenol moiety in addition to the B ring, possess this property.

The association of the diterpenes and their ability to scavenge free radicals is the key aspect in the antioxidant activity of rosemary. According to research, the antioxidant properties of the diterpenes in rosemary are described. The conjugation of the three rings and the aromatic ring (C_11_‐C_12_) in the catechol group is the key element of diterpene structure. The catechol group is responsible for scavenging the radical electron formed as a result of oxidation. The presence of three rings gives the backbone, which helps in delocalization of the charge. This conjugation is further strengthened by the presence of the carboxylic moiety (in the case of carnosic acid). The lipophilic nature of abietane diterpenoids such as carnosic acid and its antioxidant activity suggest that its functioning is similar to *α‐*tocopherol in scavenging free radicals formed as a result of the photosynthetic activity of chloroplasts [13].

## 10 Semisynthesis of Abietane Diterpenes

Most abietane diterpenoids are naturally rare; this is why much attention has been paid to their semisynthesis from readily available precursors such as carnosic acid and carnosol. These naturally occurring diterpenes are very suitable starting materials for creating structurally diverse abietane derivatives that show higher biological activity.

For instance, carnosic acid is capable of undergoing various chemical modifications due to the presence of the carboxylic acid functionality on C_20_, phenolic hydroxyl functionalities on C_11_ and C_12_, and an active benzylic position at C_7_.

Carnosol (**2**) can be quantitatively prepared from an acetone solution of carnosic acid (**1**) in the presence of molecular oxygen. Rosmanol (**3**) was the only compound formed from the reaction of the acetone solution of carnosol (**2**) with aqueous sodium bicarbonate at room temperature to introduce the oxygen function at C_7_ (Figure 7). Moreover, the catechol function was oxidized in situ to rosmaquinone (**6**) when the above reaction was carried out with a mixture of sodium bicarbonate and hydrogen peroxide (Figure 7). The semisynthetic rosmanol (**3**) and rosmaquinone (**6**) showed identical physical and spectral properties to the natural compounds as reported in the literature. The 7‐methoxyrosmanol (**5**) was the main product obtained from the reaction of carnosol (**2**) with sodium methoxide in methanol, while 7‐ethoxyrosmanol (**4**) was the main product obtained from the reaction of carnosol with sodium ethoxide in ethanol (Figure 7).

**FIGURE 7:**
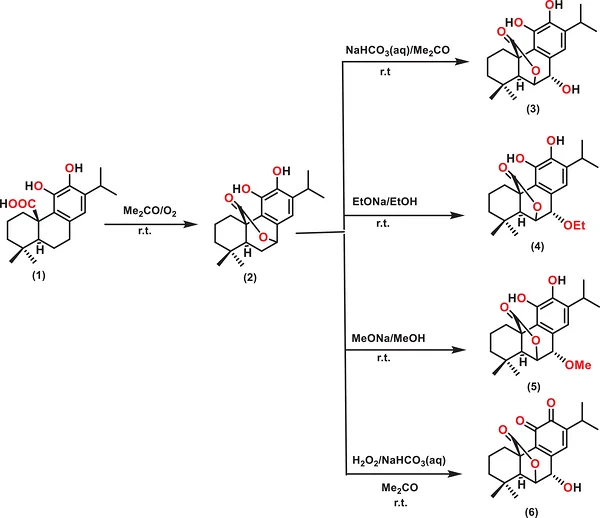
Modification of carnosic acid into derivatives using various reagents.

Pyridinium chlorochromate oxidized rosmanol (**3**), which was isolated from carnosol (**2**), yielding two compounds. Upon separation by column chromatography, these compounds were characterized as rosmaquinone (**6**) and galdosol (**7**), respectively (Figure 8). Through the less hindered si face of the carbonyl group by attacking C_7_ with a hydride, the reduction of galdosol by sodium borohydride in methanol resulted in the formation of epirosmanol (**8**) (Figure 8) as expected. In all aspects, these compounds' spectroscopic and physical properties corresponded to those of actual data and natural samples [49].

**FIGURE 8:**
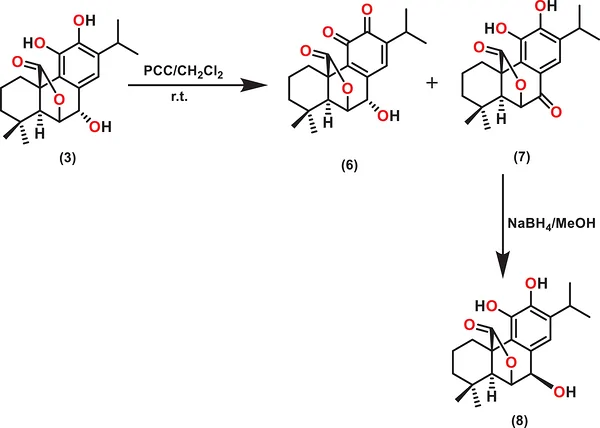
Chemical transformation of rosmanol into *epi‐rosmanol*.

By introducing certain modifications at the C‐ring and C_20_ position, a research group produced various carnosic acid derivatives (**8a–8f**) (Figure 9). The antiproliferative activity of these compounds towards the P388 murine leukaemia cell line was evaluated. Amongst these two compounds, **8a** and **8b**, with IC_50_ values of 0.27 and 0.22 µg/mL, respectively, having an ester functional group at C_20_ and an *o‐*quinone or *o*‐quinone equivalent system in the C‐ring, proved to be highly active. The results obtained were much more encouraging than those obtained with carnosic acid (IC_50_ = 1.2 µg/mL). The low effectiveness of derivative **8c** (IC_50_ = 0.6 µg/mL), which differs from the other derivatives in only one feature, implies that the presence of an *o‐*quinone group (or its analogues) in the C‐ring is necessary for the antiproliferative effect. This can be seen in the case of derivatives **8d** and **8e**, where the IC_50_ of derivative **8e** (0.94 µg/mL) became lower than that of derivative **8d** (1.9 µg/mL) because of the *o‐*quinone group added to derivative **8e** [50].

**FIGURE 9:**
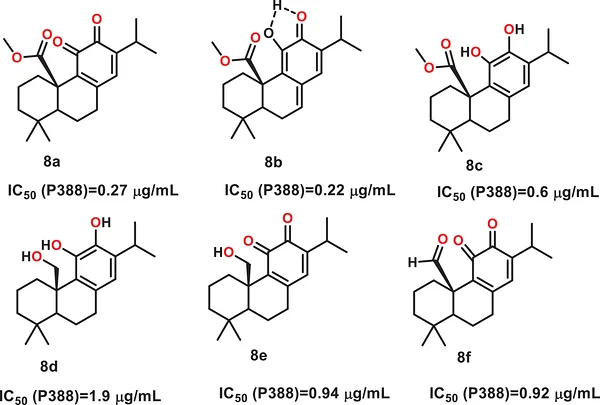
Murine leukaemia cell line P388 was employed to determine the IC_50_ values of derivatives **8a–8f** of Carnosic acid. Cells were treated with increasing concentrations of drugs for 48 h, after which IC_50_ values were determined by MTT assay.

With its potent antioxidant, anti‐inflammatory, and anticancer activities, carnosic acid has become a principal scaffold in recent semisynthetic studies in medicinal chemistry. The C_20_ carboxylic acid, catechol hydroxyls at C_11_/C_12_, and aromatic C‐ring have all undergone various modifications to generate several derivatives. These modifications include esterification, amidation, carbamate formation, acetylation, oxidation, and nitrogen substitution. These modifications were aimed at maintaining the unique abietane skeleton intact while improving lipophilicity, metabolic stability, cellular uptake, and selectivity toward targets [51, 52, 53, 54, 55].

In general, the semisynthesis of rosemary abietane diterpenoids has advanced beyond the synthesis of naturally occurring minor metabolites to the rational production of derivatives that are optimized pharmaceutically. The chemical diversity of rosemary diterpenoids has expanded greatly through these semisynthesis techniques, making the abietane scaffold a desirable starting point for future drug discovery.

## 11 Pharmacokinetics of Abietane Diterpenoids From *S. rosmarinus*

The pharmacokinetic characteristics of rosemary abietane diterpenoids have mainly been studied using carnosic acid and its metabolite carnosol in rat models. Carnosic acid was administered intragastrically (90 mg/kg), showing a time of maximum concentration (T_max_) of 125.6 min, maximum plasma concentration (C_max_) of 126 µM, area under the curve (AUC_0–t_) of 21 755.3 mg/L/min, and oral bioavailability of 65.09% in male Sprague–Dawley rats [56]. In a second study that used a lower dose of carnosic acid (64.3 mg/kg) administered intragastrically, a T_max_ of 136.6 min, C_max_ of 105 µM, AUC_0–t_ of 7.05 mg/mL/min, and oral bioavailability of 40.1% were observed [10]. The third study used rosemary extract standardized to 40% carnosic acid content (571 mg/kg rosemary extract containing 230 mg/kg carnosic acid) in female Zucker rats. A total of 26 diterpenes, including the primary metabolites, were identified, with the most abundant being carnosol, carnosic acid, rosmariquinone, carnosic acid 12‐methylether, carnosic acid glucuronide, and carnosol glucuronide. In this study, the T_max_ of carnosol is 13.3 min, its C_max_ is 18.2 µM, and its AUC_all_ is 137.4 µM h, while that of carnosic acid is 24 min, 26.6, and 178.8 µM [9]. Compared to other phytochemicals from the diet, carnosic acid is known for having a good oral bioavailability (40%–65%) that enables it to produce pharmacological effects in cellular models.

## 12 Pharmacological Activity of Abietane Diterpenoids

The secondary metabolites present in rosemary, such as carnosol, carnosic acid, etc., have drawn interest for their possible therapeutic use [57]. Abietane diterpenoids show a range of interesting biological effects. Below is a list of the most important diterpenoids and their characteristics. Here, we provide a detailed overview of some biological activities of abietane diterpenoids based on various studies.

### 12.1 Antioxidant Activity

The abietane diterpenoids in *S. rosmarinus*, including carnosic acid, carnosol, and rosmanol, contribute mainly to its antioxidant effect through their ability to scavenge free radicals and inhibit oxidative propagation reactions. This is achieved due to the phenolic and quinoid nature of these compounds, which allows them to act as chain‐breaking antioxidants [58, 59]. In addition, these molecules can chelate transition‐metal ions, especially Fe^2+,^ thereby minimizing the formation of reactive oxygen species (ROS). In contrast to synthetic antioxidants such as butylated hydroxyanisole (BHA), butylated hydroxytoluene (BHT), and propyl gallate, carnosic acid and carnosol exhibit a stronger inhibitory effect on membrane lipid peroxidation and serve as potent scavengers of peroxyl radicals.

It is reported that rosmanol shows very high antioxidant activity; in both linoleic acid and lard, it is about four times more efficient than certain commercially available antioxidants. Also, 90% of the total antioxidant power of rosemary extract is due to carnosic acid and carnosol, which act effectively as scavengers of hydroxyl and peroxyl radicals, preventing lipid peroxidation in biological membranes. Thus, the use of abietane diterpenoids from rosemary as natural antioxidants is medically significant [60, 61].

### 12.2 Antimicrobial Activity

Abietane diterpenes are key phytochemical components of *S. rosmarinus* that contribute significantly to its antimicrobial effect. Research into these compounds has identified several specific diterpenoids with varying degrees of antibacterial potency against both Gram‐positive and Gram‐negative bacteria [62, 63].

Studies have identified various abietane‐type diterpenoids in rosemary extracts. 12‐methoxy‐carnosic acid exhibits significant inhibitory effects against several strains. It has been shown to have a Minimum Inhibitory Concentration (MIC) of 8 µg/ml against *Staphylococcus aureus*, 16 µg/ml against *Bacillus subtilis*, and 32 µg/ml against *Pseudomonas aeruginosa*. Galdosol, another potent abietane diterpene, shows strong activity with MIC values of 16 µg/ml against *S. aureus* and 32 µg/ml against both *B. subtilis* and *P. aeruginosa* [62]. Moreover, carnosic acid and carnosol are frequently credited with antibacterial effects against pathogens such as *S. aureus, Escherichia coli*, etc [64]. Utilizing these naturally occurring diterpenes may offer a therapeutic alternative to synthetic antibiotics, potentially addressing issues related to increasing bacterial resistance [63].

### 12.3 Anti‐Inflammatory Activity

The activity of nuclear factor kappa‐light‐chain‐enhancer of activated B cells (NF‐*K*B) was constrained by rosemary and its hexane fractions through inhibiting the phosphorylation of mitogen‐activated protein kinases (MAPKs). As a result, there was a decrease in the synthesis of two key enzymes, cyclooxygenase‐2 (COX‐2) and inducible nitric oxide synthase (iNOS), which are involved in inflammatory processes through the generation of NO and PGE2, respectively [65]. This was consistently seen in another study that used extracts of fresh rosemary leaves and their purified components, carnosol and CA, on mouse skin (Figure 10) [66].

**FIGURE 10:**
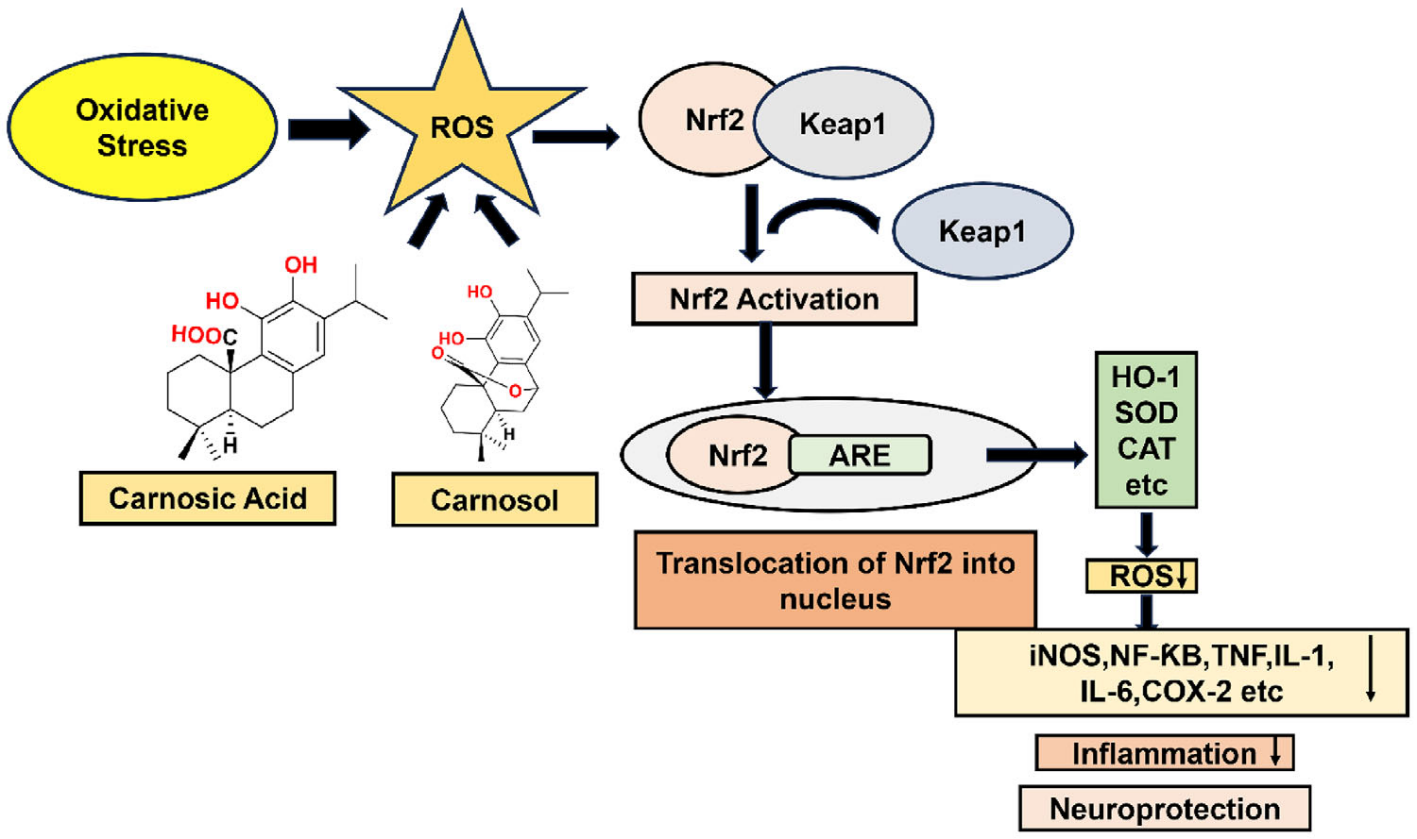
The effect of Carnosic acid and Carnosol on inflammation and neuroprotection.

### 12.4 Neuroprotective Activity

Rosemary produces a profound therapeutic effect on mood, anxiety, pain, learning, and sleep. Nervous system disorders are characterized by abnormalities in the central or peripheral nervous system structure or function. Trauma, infections, metabolic diseases, or hereditary conditions may trigger these diseases. In conventional medicine, rosemary has been utilized to cure numerous diseases, including dysmenorrhea, epilepsy, rheumatic pain, spasms, hysteria, and physical and mental fatigue (Figure 10).

The Nuclear factor erythroid 2‐related factor 2/Kelch‐like ECH‐associated protein 1 (Nrf2/Keap1) signaling pathway acts as the primary process that mediates the neuroprotective effects of carnosic acid and carnosol. The abietane diterpenoids help Nrf2 dissociate from Keap1 when there is oxidative stress, allowing Nrf2 to enter the nucleus and bind to antioxidant response elements (AREs). As a result, the intracellular reactive oxygen species (ROS) levels inside the cell are decreased, and the redox balance is restored due to the increased synthesis of protective enzymes, including heme oxygenase‐1 (HO‐1), superoxide dismutase (SOD), and catalase (CAT).

The resultant reduction in oxidative stress leads to the prevention of neuroinflammation and neuronal damage by inhibiting the generation of inflammatory mediators such as NF‐*K*B, Tumor Necrosis Factor‐alpha (TNF‐*α*), Interleukin‐1 beta (IL‐1*β)*, Interleukin‐6 (IL‐6), iNOS, and COX‐2. Carnosic acid and carnosol protect against neurodegeneration through the synergistic regulation of oxidative and inflammatory signaling pathways.

Parkinson's disease is an age‐associated disorder that leads to the degeneration of certain areas of the brain. It is well‐known for producing tremors, slow movements, and balance problems. The chief constituent of *S. rosmarinus*, carnosol, possesses highly active anti‐inflammatory and antioxidant properties. A study revealed that carnosol safeguarded the cultured dopaminergic neuronal cell line (SN4741) against rotenone‐induced neurotoxicity. Furthermore, carnosol safeguarded cultured dopaminergic neuronal cells (SN4741) from dieldrin‐induced neurotoxicity. The neuroprotective actions of carnosol are attributed to the Raf‐MEK‐ERK1/2 pathways (Raf‐mitogen‐activated protein kinase (MEK)‐extracellular signal‐regulated kinase (ERK)1/2 signaling pathway), which block cell death in cells of dopamine neurons and increase tyrosine hydroxylase expression. As an antioxidant, carnosol can be a potential therapy for Parkinson's disease symptoms as well as the dopamine system [67, 68].

### 12.5 Anticancer Activity

The uncontrolled growth of abnormal cells that possess the ability to invade and kill normal cells in the body is referred to as cancer. The second leading cause of death globally is cancer. Nonetheless, due to advances in the detection of cancer, treatment of cancer, and prevention of cancer, survival rates for many types of cancer are increasing. Rosemary extracts have been shown to possess a wide variety of anticancer properties against many forms of cancer. Nevertheless, in vitro and animal experiments have demonstrated the anticancer activities of rosemary, some of which are attributed to its primary components, including carnosic acid and carnosol. Ethanolic rosemary extract was able to prevent DNA damage in CaCo‐2 colon carcinoma cells caused by hydrogen peroxide and visible light‐excited methylene blue. Based on its free radical scavenging ability, the use of rosemary in colon cancer prevention was established. Methanolic, ethanolic, and supercritical rosemary extracts were employed in a study to establish the antiproliferative activity of rosemary on breast cancer cells. The most active compound in this activity was carnosic acid in many of these studies. From the many extraction methods, rosemary's bioactive compounds were best extracted with supercritical fluids compared to methanolic extraction, and these extracts reliably proved to be most inhibitory to cell proliferation [69].

The anticancer activity of bioactive components of *Salvia rosmarinus*, carnosic acid and carnosol, is characterized by a multifaceted mechanism of action that targets various stages of tumor development and progression (Figure 11).

**FIGURE 11:**
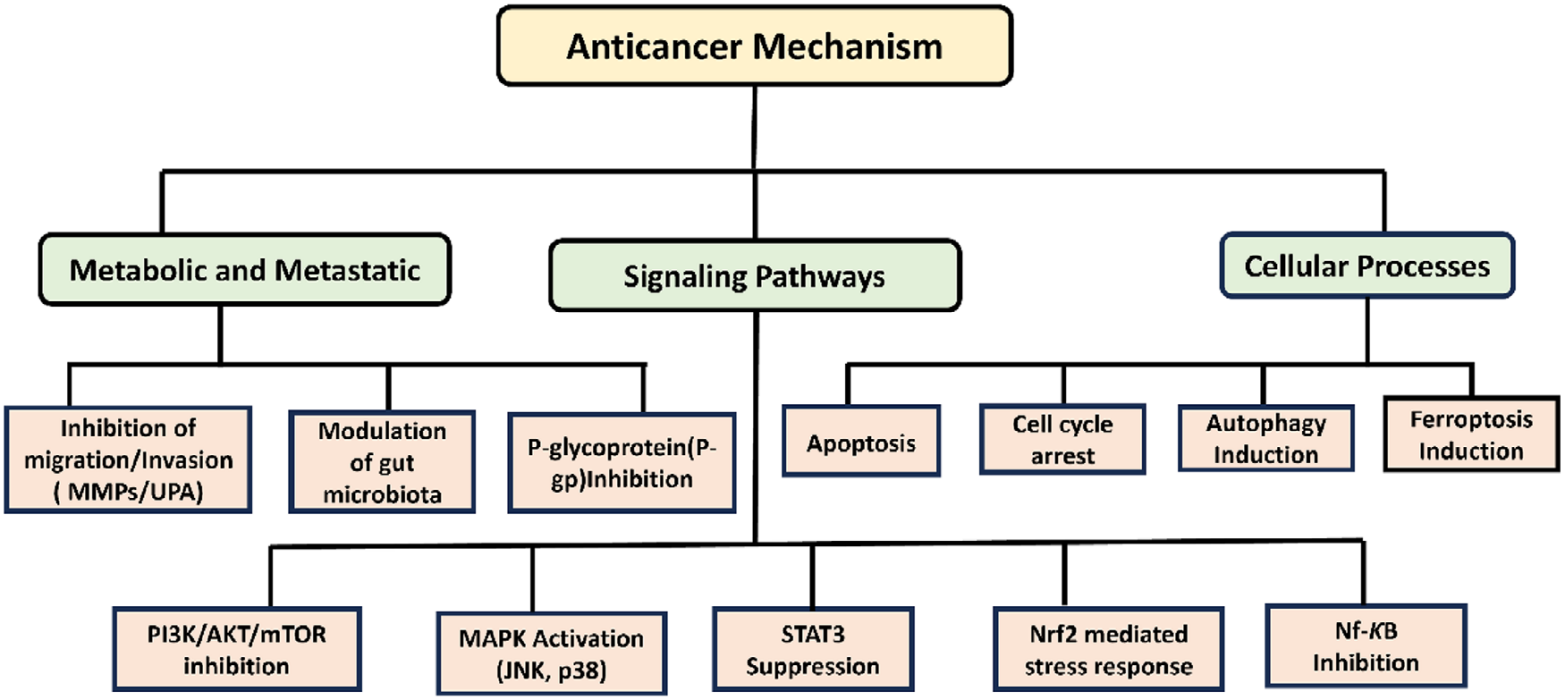
Mechanism of anticancer activity of abietane diterpenes.

The general descriptive mechanism of action includes the following processes:

#### 12.5.1 Induction of Apoptosis

*S. rosmarinus* compounds trigger programmed cell death through both the intrinsic (mitochondrial) and extrinsic (death receptor) pathways [70]. This process involves:

##### 12.5.1.1 Modulation of Pro/Anti‐Apoptotic Proteins

Upregulation of pro‐apoptotic markers such as Bcl‐2‐associated X protein (Bax) and p53, and the downregulation of anti‐apoptotic proteins like *β*‐cell lymphoma‐2 (Bcl‐2), survivin, and mouse double minute 2 (Mdm2) [71].

##### 12.5.1.2 Caspase Activation

*S. rosmarinus* compounds lead to the activation of the caspase cascade (e.g., caspases‐3,7 and 9) and the subsequent cleavage of Poly(ADP‐ribose) polymerase (PARP), which are hallmarks of irreversible cell death [72].

#### 12.5.2 Cell‐Cycle Arrest

The bioactive compounds of *S. rosmarinus* inhibit tumor cell proliferation by arresting the cell cycle at various phases, most commonly G_0_/G_1_, S, or G_2_/M phases, depending on the cancer type. This is achieved by:

1. Inhibiting cyclins and cyclin‐dependent kinases (CDKs): Reducing the levels of cyclins and CDKs is necessary for phase transition.
2. Upregulating inhibitors: Increasing the expression of CDK inhibitors such as p21WAF1 and p27KIP1 [73].

#### 12.5.3 Modulation of Key Signaling Pathways

*S. rosmarinus* diterpenes disrupt several signaling cascades essential for cancer cell survival and growth.

1. Phosphoinositide 3‐kinase (PI3K)/protein kinase B (AKT)/ mechanistic target of rapamycin (mTOR) pathway: Inhibition of this pathway leads to decreased protein synthesis and survival signals.
2. MAPK pathways: *S. rosmarinus* modulates extracellular signal‐regulated kinase (ERK1/2), c‐Jun N‐terminal kinase (JNK), and p38 pathways, which are critical for mediating apoptosis and stress responses [74, 75].
3. Janus kinase (JAK)/ Signal Transducer and Activator of Transcription (STAT) Pathway: Compounds like CA and CS inhibit STAT3 and STAT5 phosphorylation, suppressing the expression of genes linked to proliferation and survival [70].
4. NF‐KB pathway: Suppression of NF‐*K*B activation reduces inflammation and the expression of survival factors [76].

#### 12.5.4 Generation of Oxidative Stress(ROS)

While *S. rosmarinus* acts as an antioxidant in normal cells by activating the Nrf2 pathway, it often functions as a pro‐oxidant in cancer cells. The generation of ROS in tumor cells leads to the following:

1. Endoplasmic reticulum (ER) stress: Triggering the unfolded protein response and mitochondrial damage.
2. Ferroptosis: Inducing cell death via iron‐dependent lipid peroxidation in specific resistant cell types [77, 78].

#### 12.5.5 Inhibition of Metastasis, Invasion, and Angiogenesis

*S. rosmarinus* impairs the ability of cancer cells to spread and form new blood vessels:

1. Anti‐invasive effects: Downregulation of proteases like matrix metalloproteinases (MMP‐2, MMP‐9) and urokinase Plasminogen Activator (uPA), while increasing tissue inhibitors of metalloproteinases (TIMPs) [75, 79].
2. Epithelial mesenchymal transition (EMT) reversal: Upregulation of the epithelial marker E‐cadherin and downregulation of mesenchymal markers like *N*‐cadherin and vimentin, effectively blocking the epithelial‐to‐mesenchymal transition [80].

#### 12.5.6 Sensitization and Synergy

*S. rosmarinus* can overcome chemoresistance by inhibiting drug efflux transporters such as P‐glycoprotein (P‐gp), thereby increasing the efficacy of chemotherapeutic agents like cisplatin, 5‐fluorouracil, and doxorubicin. It also exhibits synergistic effects when combined with various drugs and natural products [77, 81].

## 13 Safety Profile and Toxicity Effects of Abietane Diterpenoids

The use of rosemary extract as a potent, safe natural antioxidant for food preservation has been authorized by the European Union [82]. While the topical and oral use of rosemary is generally considered safe, it is imperative to note that herbal medicines can also produce negative reactions. Rashes and allergies could result from rosemary formulations used by some individuals. Moreover, some groups, such as those with kidney or liver ailments, and pregnant and lactating women, should be careful when using rosemary [7, 83].

Although abietane diterpenoids from *S. rosmarinus*, such as carnosic acid, carnosol, and rosmanol, possess remarkable pharmacological features, their safety profile should be considered while developing their use in medicine. In vitro studies using differentiated and undifferentiated HepaRG cells demonstrated that carnosic acid, carnosol, and rosmanol proved to possess relatively lower toxicity towards differentiated cells owing to their higher detoxifying potential. Rosmanol induced the most substantial metabolic change, followed by carnosol and carnosic acid [84].

The acute and 30‐day oral toxicity of carnosic acid has been studied in an in vivo experiment. In the acute toxicity test, the median lethal dose (LD_50_) of carnosic acid in mice was found to be 7100 mg/kg. Histopathology alteration was seen in the heart, liver, and kidneys of surviving mice after administering a single dose of carnosic acid. In the subchronic toxicity test, administration of carnosic acid in rats for 30 days led to a slight reduction in weight gain pattern, but not considerably in comparison with the control group. High and moderate doses of carnosic acid produced elevated levels of aminotransferase (AST) but low levels of total serum protein in the blood biochemical examination. According to histopathology analysis, rats treated with high doses of carnosic acid had minor pathological abnormalities in their liver, kidney, and heart [85].

However, based on the currently available information, it seems that the abietane diterpenoids found in *S. rosmarinus* have a good safety margin even when used in therapeutic dosages. However, more detailed studies regarding their chronic toxicity, reproductive safety, pharmacokinetics, and clinical tests in humans should be carried out.

## 14 Future Perspective

The usefulness of abietane‐type diterpenoids isolated from *S. rosmarinus* as natural medicines is reflected by the growing evidence supporting their therapeutic potential. In view of proving efficacy and safety in humans, future studies should focus on translating the accumulated body of data from in vitro experiments and animal models to clinical trials in humans. Given the ability of carnosic acid and carnosol to cross the blood‐brain barrier and affect oxidative and inflammatory pathways via activation of the Nrf2/Keap1 pathway, their use for neuroprotection deserves special attention. Valuable diterpenoids such as carnosic acid, carnosol, rosmanol, and epirosmanol can be produced sustainably owing to advances in metabolic engineering, synthetic biology, and plant biotechnology.

In addition, the stability, bioavailability, and tissue‐specific targeting can be increased through the use of liposomal delivery systems, nanoformulations, and other drug‐delivery techniques. In addition, more potent derivatives, which will demonstrate superior antitumor, anti‐inflammatory, antibacterial, and neuroprotective effects, can be developed through further study of structure‐activity relationships and semi‐synthetic modifications. Besides studying the currently available diterpenoids, there are other types of diterpenoids and their activities that need to be explored. Additionally, the entire use of *S. rosmarinus* will eventually become a focus of research.

## 15 Conclusion

The abietane‐type diterpenoids, which comprise carnosic acid, carnosol, rosmanol, epirosmanol, isorosmanol, and galdosol found in *S. rosmarinus*, have been established to be a highly diverse group of natural compounds with great pharmaceutical value. The occurrence, biochemical pathway through geranylgeranyl diphosphate, diversity in structure, metabolism, semi‐synthesis, pharmacology, pharmacokinetics, and several biological functions of these compounds have been covered in this review. Of these compounds, carnosic acid is one of the most important compounds that plays the role of a pharmacologically active compound and also forms various oxidized abietane derivatives with numerous properties.

Although semisynthesis strategies have proven that targeted modifications of the abietane skeleton could lead to the enhancement of their biological activities, the latest advancements in green extraction approaches have increased the efficiency in isolating such bioactive diterpenoids. The problem of transforming the rosemary abietane diterpenoids into clinically viable drugs remains unresolved due to poor standardization, lack of human trials, and insufficient knowledge of their mechanisms of action despite positive preclinical findings. In any case, it should be mentioned that the plant *S. rosmarinus* represents an environment‐friendly source of biologically active abietane diterpenoids, and future investigations could be expected.

## Author Contributions

**Rida Siddiqui**: conceptualization, methodology, formal analysis, data curation, writing – original draft. **Vipin Singh**: writing – original draft; **Kapil Dev**: conceptualization, methodology, resources, supervision, writing – original draft, writing – review and editing.

## Funding

The authors received funding support from the Council of Scientific and Industrial Research (CSIR) Aroma Mission (HCP‐0007 and MMP‐025304) and CSIR‐FTT020502/CRM1 projects.

## Conflicts of Interest

The authors declare no conflicts of interest.

## Data Availability

Data sharing is not applicable to this article, as no datasets were generated or analyzed during the current study.
